# Successful blood glucose management of a severe COVID-19 patient with diabetes: A case report

**DOI:** 10.1097/MD.0000000000020844

**Published:** 2020-06-26

**Authors:** Rujun Hu, Huiming Gao, Di Huang, Deyu Jiang, Fang Chen, Bao Fu, Xiaoli Yuan, Jin Li, Zhixia Jiang

**Affiliations:** a Department of Emergency; b Department of Nursing; c Department of Critical Care Medicine, Affiliated Hospital of Zunyi Medical University, Zunyi, Guizhou, China.

**Keywords:** blood glucose, coronavirus disease 2019, diabetes, novel coronavirus, severe acute respiratory syndrome coronavirus 2

## Abstract

**Rationale:**

Coronavirus disease 2019 (COVID-19) has emerged as a rapidly spreading communicable disease affecting individuals worldwide. Patients with diabetes are more vulnerable to the disease, and the mortality is higher than in those without diabetes. We reported a severe COVID-19 patient with diabetes and shared our experience with blood glucose management.

**Patient concerns:**

A 64-year-old female diabetes patient was admitted to the intensive care unit due to productive coughing for 8 days without any obvious cause. The results of blood gas analysis indicated that the partial pressure of oxygen was 84 mm Hg with oxygen 8 L/min, and the oxygenation index was less than 200 mm Hg. In addition, postprandial blood glucose levels were abnormal (29.9 mmol/L).

**Diagnoses:**

The patient was diagnosed with COVID-19 (severe type) and type 2 diabetes.

**Interventions:**

Comprehensive interventions including establishing a multidisciplinary team, closely monitoring her blood glucose level, an individualized diabetes diet, early activities, psychological care, etc, were performed to control blood glucose while actively treating COVID-19 infection.

**Outcomes:**

After the comprehensive measures, the patient's blood glucose level gradually became stable, and the patient was discharged after 20 days of hospitalization.

**Lessons:**

This case indicated that the comprehensive measures performed by a multidisciplinary team achieved good treatment effects on a COVID-19 patient with diabetes. Targeted treatment and nursing methods should be performed based on patients’ actual situations in clinical practice.

## 1 Introduction

Coronavirus disease 2019 (COVID-19) is caused by severe acute respiratory syndrome coronavirus 2, and it has emerged as a rapidly spreading communicable disease affecting individuals worldwide. On March 19, 2020, there were 209,839 confirmed cases (81,174 in China and 128,665 outside of China), and COVID-19 has led to 8778 deaths globally,^[1]^ which has posed a serious threat to human safety.

The population is generally susceptible to this coronavirus, especially those with diabetes who are more vulnerable to the disease.^[2]^ Several published studies have reported that 12% to 22% of COVID-19 patients have comorbid diabetes,^[3–7]^ and a report of 72,314 patients with COVID-19 published by the Chinese Center for Disease Control and Prevention found increased mortality in people with diabetes (2.3% overall and 7.3% patients with diabetes).^[8]^ In addition, Zhou's research results also revealed that the risk of in-hospital death was 2.85 times higher in patients with diabetes than in those without diabetes.^[7]^ Obviously, the presence of diabetes is associated with increased mortality. Therefore, the blood glucose management of diabetic patients plays an important role in reducing mortality. This case presentation describes a successful experience with blood glucose management in a severe COVID-19 patient with diabetes.

## 2 Case presentation

A 64-year-old woman with COVID-19 was admitted to our hospital on February 19, 2020, due to productive coughing for 8 days without any obvious cause. The severe acute respiratory syndrome coronavirus 2 RNA test was positive, and a chest computerized tomography scan revealed that both lungs had multilobe patchy density with an increased shadow, infectious lesions, suspicious viral pneumonia (lung involvement +++), and pneumonectasis. The heart was enlarged, predominantly in the left ventricle, the aorta was hardened, and mediastinal lymph nodes were enlarged. A physical examination at admission revealed a body temperature of 36.7°C, a pulse of 90 beats per minute, a respiratory rate of 20 breaths per minute, a blood pressure of 110/70 mm Hg, and an arterial oxygen saturation of 90% with oxygen of 7 L/min. The breath sounds of both lungs were thick with wet rales. Initial laboratory tests showed a white blood cell count of 7.4 × 10 ^9^/L, neutrophil ratio of 77.2%, and lymphocyte ratio of 13.4%. Urine sugar + + + +, and acetone bodies ++. She was diagnosed with COVID-19 (severe infection) and type 2 diabetes. Lopinavir and ritonavir were given orally for antiviral therapy as well as interferon alpha inhalation. On the second day after admission, the biochemical test revealed multiple abnormal results (blood glucose 17.4 mmol/L, interleukin-6 61.31 pg/mL, erythrocyte sedimentation rate 75 mm/h, etc). The results of blood gas analysis indicated that the partial pressure of oxygen was 84 mm Hg with oxygen 8 L/min, and the oxygenation index was less than 200 mm Hg. Therefore, she was transferred to the intensive care unit on February 20, 2020 with the diagnosis of COVID-19 (critical infection) and type 2 diabetes and high-flow oxygen therapy was given (no mechanical ventilation). On the second day of intensive care unit admission, the patient's vital signs were relatively stable except for abnormal postprandial blood glucose (29.9 mmol/L) at 17:30, and the peripheral blood glucose level was 22.2 mmol/L at 18:30. Insulin, 500 mL saline + insulin 25 IU, was used to control blood glucose, and the transfusion speed was adjusted according to the level of peripheral blood glucose. Based on the patient's disease situation, we implemented comprehensive measures to control blood glucose while actively treating COVID-19 infection.

First, we established a multidisciplinary team, including specialists in critical care, respiratory, endocrinology, and nursing, and discussed treatment and care plans on a daily basis. Given the postprandial and nocturnal blood glucose of the patient were relatively high, and oral hypoglycemic agents could not be effective in controlling the patient's blood glucose, therefore, insulin was used for the patient. Based on the current blood glucose condition, endocrine experts recommended a predinner subcutaneous injection of 5 to 15 IU insulin Hspart and 10 to 28 IU insulin glargine injection before sleeping. The dose of insulin was adjusted daily according to the patient's blood glucose to maintain a fasting glucose level 6 to 8 mmol/L and a postprandial blood glucose level of 8 to 14 mmol/L.

Second, an individualized diabetic diet plan was formulated for the patient by dietitians and endocrinologists to degrade the blood glucose fluctuation caused by food.

Third, medical staff tailored an early activity plan for the patient, which mainly included the following 3 aspects:

a. body position management. Nurses assisted the patient in changing body position, such as sitting on the bed, sitting beside the bed, and standing on the ground. Postural training lasted 30 minutes per session, 2 times a day, within the patient's tolerance;
b. activity management. During hospitalization, we conducted early activities for the patient, which included passive movement and active motion. The total training time was less than 30 minutes per session and was limited to not causing aggravating fatigue; and
c. respiratory management. A vibration sputum apparatus was used to promote sputum discharge. In addition, abdominal breathing and blowing balloons were also performed to exercise the patient's breathing function.

Fourth, the patient was assisted in maintaining good mental health.

(a) The patient's psychological status was evaluated, and the root of the psychological problems was identified through communication with the patient.
(b) The patient was assisted with establishing a social support system by the following methods. We strengthened the contact between the patient and family members by WeChat, telephone, etc, to encourage her. In addition, college students in a medical university were encouraged to write letters containing blessings and encouragement for the patient so that she could feel care from society. In addition, a WeChat group of patients who needed mutual assistance was set up, which was managed by specialized medical staff who could solve the problems patients faced in a timely manner, and patients could share their successful experiences with each other as well.
(c) Virtual reality technology was applied. We made full use of immersive, interactive, and imaginative virtual reality technology and put the patient in a virtual environment to eliminate her negative emotions.
(d) The patient's attention was diverted. An iPad and a small stereo were used to play videos and soothing music that the patient liked to help her recall good memories before the illness, to reduce the time spent immersed in thoughts about the ward or illness, and to relieve anxiety and depression.

Fifth, we monitored blood glucose changes closely according to 7 time points (before and 2 hours after 3 meals, before sleep and during the night). We designed a blood glucose monitoring form to record the results after each measurement and told the patient the specific blood glucose value every time, aiming to remind her to pay attention.

**Figure 1:**
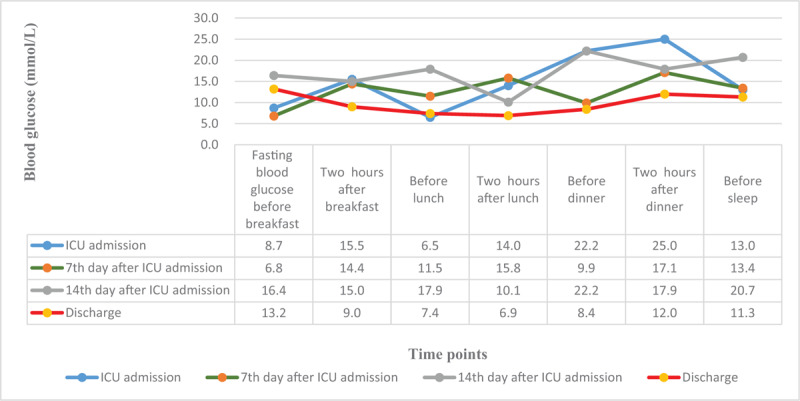
Changes trends of blood glucose in the patient. This figure showed that the patient's blood glucose was high and fluctuated significantly at the time of intensive care unit admission, on the 7th and 14th day after intensive care unit admission, and that the blood glucose almost returned to normal level when discharged.

After implementing the comprehensive measures mentioned above without adverse or unanticipated events, the patient's blood glucose gradually became stable (Fig. 1). She was discharged from the hospital on March 10, 2020, after 2 consecutive negative nucleic acid tests for novel coronavirus, normal blood glucose levels and achieving a satisfactory condition. After the patient was discharged from the hospital, she was followed up by professional medical staff to monitor her blood glucose fluctuations and COVID-19 related condition, and telemedicine (WeChat) was used for remote guidance.

The Ethics Committee of the Affiliated Hospital of Zunyi Medical University (Guizou, China) approved the study protocol, and informed consent was obtained from the patient for publication of this case report.

## 3 Discussion

To our knowledge, this is the first report to share experience with blood glucose management in a severe COVID-19 patient with diabetes. The patient recovered and was discharged after 20 days of hospitalization by comprehensive treatment and nursing measures.

Data about COVID-19 in patients with diabetes are limited at present. Kulcsar's research revealed that diabetes was associated with greater weight loss and greater pulmonary inflammation.^[9]^ Another study published by the Chinese Center for Disease Control and Prevention also showed that the overall mortality of COVID-19 patients was 2.3%, while the number was 7.3% in those patients with diabetes.^[8]^ It is obvious that diabetics have a higher risk of lung infection and death than their counterparts. Meanwhile, blood glucose control in COVID-19 patients with diabetes is difficult because of stress and anxiety condition,^[2,10]^ pancreatic tissue as a potential target for the virus,^[11]^ and the inability for quarantined patients to perform exercise due to space constraints or poor pulmonology condition.^[12]^ Therefore, great attention should be paid to controlling patients’ blood glucose levels to reduce the complications and mortality caused by diabetes.

For this patient, we established a multidisciplinary team and closely monitored her blood glucose level and formulated a series of measures, including an individualized diabetes diet, early activities, psychological care, etc. Obviously, the measures undertaken resulted in the desired effects. In the process of treating and caring for COVID-19 patients with diabetes, it is necessary to pay attention to the following issues. First, when patients’ blood glucose fluctuates greatly, it is recommended to report to an endocrinologist before injecting insulin to determine whether the insulin dose needs to be adjusted. Second, diabetes diets are different from patients’ daily eating habits. Therefore, it is extremely important to perform good health education and to obtain the patients’ support and cooperation in order to enhance their compliance. Third, early activities should be carried out step by step according to patients’ individual situations and should be implemented while ensuring patient safety. Fourth, in regard to psychological care, it is important to understand patients’ family backgrounds, educational levels, hobbies and other information. Only by knowing patients’ basic information can medical staff implement individualized and targeted psychological care.

## 4 Conclusion

This case showed that the comprehensive measures performed by a multidisciplinary team achieved good treatment effects on a COVID-19 patient with diabetes. Targeted treatment and nursing methods should be performed based on patients’ actual situations in clinical practice.

## Author contributions

**Investigation:** Rujun Hu, Xiaoli Yuan, Fang Chen, Jin Li and Di Huang.

**Writing – original draft:** Rujun Hu, Huiming Gao, and Bao Fu.

**Writing – review & editing:** Zhixia Jiang and Deyu Jiang.
